# Significance of Immune Status of SARS-CoV-2 Infected Patients in Determining the Efficacy of Therapeutic Interventions

**DOI:** 10.3390/jpm12030349

**Published:** 2022-02-25

**Authors:** Ganesh Dattatraya Saratale, Han-Seung Shin, Surendra Krushna Shinde, Dae-Young Kim, Rijuta Ganesh Saratale, Avinash Ashok Kadam, Manu Kumar, Ali Hassan Bahkali, Asad Syed, Gajanan Sampatrao Ghodake

**Affiliations:** 1 Department of Food Science and Biotechnology, Dongguk University-Seoul, 32 Dongguk-ro, Ilsandong-gu, Goyang-si 10326, Gyeonggi-do, Korea; gdsaratale@dongguk.edu (G.D.S.); spartan@dongguk.edu (H.-S.S.); 2 Department of Biological and Environmental Science, Dongguk University-Seoul, 32 Dongguk-ro, Ilsandong-gu, Goyang-si 10326, Gyeonggi-do, Korea; shindesurendra9@gmail.com (S.K.S.); sbpkim@dongguk.edu (D.-Y.K.); 3 Research Institute of Biotechnology and Medical Converged Science, Dongguk University-Seoul, 32 Dongguk-ro, Ilsandong-gu, Goyang-si 10326, Gyeonggi-do, Korea; rijutaganesh@gmail.com (R.G.S.); avikadam2010@gmail.com (A.A.K.); 4 Department of Life Science, Dongguk University-Seoul, 32 Dongguk-ro, Ilsandong-gu, Goyang-si 10326, Gyeonggi-do, Korea; manukumar007@gmail.com; 5 Department of Botany and Microbiology, College of Science, King Saud University, P.O. Box 2455, Riyadh 11451, Saudi Arabia; abahkali@ksu.edu.sa (A.H.B.); assyed@ksu.edu.sa (A.S.)

**Keywords:** coronavirus, SARS-CoV-2, immune response, therapeutic interventions, immunopathogenesis

## Abstract

Coronavirus disease 2019 (COVID-19) is now being investigated for its distinctive patterns in the course of disease development which can be indicated with miscellaneous immune responses in infected individuals. Besides this series of investigations on the pathophysiology of severe acute respiratory syndrome coronavirus 2 (SARS-CoV-2), significant fundamental immunological and physiological processes are indispensable to address clinical markers of COVID-19 disease and essential to identify or design effective therapeutics. Recent developments in the literature suggest that deficiency of type I interferon (IFN) in serum samples can be used to represent a severe progression of COVID-19 disease and can be used as the basis to develop combined immunotherapeutic strategies. Precise control over inflammatory response is a significant aspect of targeting viral infections. This account presents a brief review of the pathophysiological characteristics of the SARS-CoV-2 virus and the understanding of the immune status of infected patients. We further discuss the immune system’s interaction with the SARS-CoV-2 virus and their subsequent involvement of dysfunctional immune responses during the progression of the disease. Finally, we highlight some of the implications of the different approaches applicable in developing promising therapeutic interventions that redirect immunoregulation and viral infection.

## 1. Introduction

In consideration of public health emergency and global reach, on 11 March 2020, the World Health Organization (WHO) specified coronavirus disease 2019 (COVID-19) as a global pandemic outbreak of international public health concern [1]. A novel, highly transmissible enveloped RNA betacoronavirus unexpectedly emerged in December 2019 in Wuhan, China, and then was formally named as severe acute respiratory syndrome coronavirus 2 (SARS-CoV-2. The most common clinical symptoms and manifestations of SARS-CoV-2 infection are pneumonia-like, including fever, hypoxia, dyspnea (labored breathing), headache, myalgia, cough, and in some cases, intestinal symptoms [2,3]. COVID-19 is now characterized as a mild to severe respiratory disease, and its clinical presentation to be influenced by comorbidities (patients with cardiovascular or renal disorders) and age factors [4]. A growing literature pointed out that the asymptomatic or mild infections are a significant fraction [5], at large half of spread events reported from pre-symptomatic and asymptomatic infections [6] and has immense implications of silent transmission. However, approximately 10–30% of SARS-CoV-2 infected patients who were hospitalized are associated with severe illness requiring intensive care to aid respiratory comfort [7,8].

The factors triggering severe disease progression in SARS-CoV-2 infected patients is yet to be wholly understood. Progression of severe immune status is not exclusively associated with the viral load but could be relevant to weak interferon response [9,10]. An excess inflammatory immune response in individuals infected by SARS-CoV-2 viruses is also known to ensure the foremost cause of severe disease development, organ failure, and mortality [11,12]. Besides this, it is interrelated with elevated levels of cytokines, mainly circulating ones [12], deep lymphopenia [13], and involved with extensive mononuclear cell infiltration in different organs, including lungs [14], spleen [15], heart [16], lymph nodes [17] and kidney [18], as observed in a post-mortem examination. Our previous report emphasized the significance of understanding the biological characteristics of the SARS-CoV-2 virus and its biomarkers applicable to developing new diagnostic kits and establishing point-of-care testing and surveillance measures [19].

The mortality concerns and morbidity-related complications realized in SARS-CoV-2 infection are associated with extensive inflammation [20]. Therefore, the need for a clear understanding of immunopathological factors underpinning the diverse responses observed in the infected patients is of paramount significance to identify appropriate therapeutic targets [21]. Several immunomodulatory agents are currently underpinning clinical trials at a rapid pace [22], and some of them are already being under routine use in the clinical practices in off-label use of drugs [23]. An in-depth understanding of each specific inflammatory pathway and cell-type shows promising results in choosing appropriate immunotherapeutic targets. Some of the recently developed strategies could be advantageous to avoid detrimental consequences in individual patients and/or during different stages of the SARS-CoV-2 infection. Presentation of specified therapeutic options, including intravenous immunoglobulin, steroid medications (methy/prednisolone, dexamethasone), personalized cytokine blockade (e.g., tocilizumab or anakinra), and JAK inhibition so far have been shown as potential alternatives in those individuals suffering from severe SARS-CoV-2 infection. In this account, we discuss potential inflammatory responses that have been identified so far in individuals with COVID-19 disease. In particular, we briefly highlight emerging pharmaceutical interventions, therapeutic modulations for pulmonary phase, inflammatory immunopathogenesis, immune-boosting strategies, the significance of tracking immune status, and specific aspects of macrophages and monocytes in the pathophysiology of SARS-CoV-2 infection.

## 2. Therapeutic Interventions

### 2.1. Pharmaceutical Interventions

The most important principle can be proactive efforts in preventing and controlling infectious diseases by reducing the source of contagion, interventions against most of the routes of spread, and protecting the susceptible old and juvenile populations. SARS-CoV-2 viruses mainly spread via respiratory aerosol droplets and rarely by surface contacts. Basic personnel and public protective measures are found to be helpful to restrict the transmission of SARS-CoV-2 viruses. At present, supportive therapies are mostly being adopted to treat symptomatic SARS-CoV-2 patients, including the treatments of other common colds, symptom aid, and complete protection. Supportive treatments are adopted to protect internal organs such as the lungs or brain, proactive prevention, emerging treatment options, pursuing individual complications, and ventilation support, if essential. Additional care should be provided to maintain the balance of electrolytes and water, thus comforting the strength of the internal organs.

Implementing vaccination programs is an effective measure to protect overall populations, but to date, the effective vaccines for SARS-CoV-2 viruses are now available in public service; some laboratories and private companies successfully completed clinical trials and distribution for vaccination. Several research institutions and private enterprises have reproposed several different technologies, including nanovaccine technology using mRNA, recombinant vaccine or inactivated vaccine, and DNA vaccine, to establish vaccine adjuncts against SARS-CoV-2 infections. For instance, a possible mRNA-based vaccine against SARS-CoV-2 viruses has been claimed by a Biotech Company called Moderna after tirelessly working in collaboration with pioneer institutes in health. AbMax Biotechnology Co., Ltd, Kechuang, China and several other private enterprises have also announced that they successfully develop vaccines against the SARS-CoV-2 viruses.

At present, few specific or compelling antiviral drugs are available to treat SARS-CoV-2 infected patients in the hospital. The discovery of new drugs, performing all steps in trials, and using them as antiviral drugs for SARS-CoV-2 viruses is a time-consuming process; instead, the most influential research strategy could be the use of old drugs. Gilead Sciences in the United States claimed that remdesivir a nucleoside analog prodrug is under development that is a broad-spectrum antiviral medication found efficient against Ebola virus [24]. Both in-vitro and in-vivo trials confirmed that even a low dose of remdesivir drug could have good inhibitory effects against the growth of both the SARS-CoV and MERS-CoV [25]. The remdesivir drug, as most potential drugs against the SARS-CoV-2 viruses, could be adopted after complete pharmacokinetic studies and completing safety protocols. A phase III clinical trial has been completed for a radcivir drug that was recently launched to treat SARS-CoV-2 infected patients by Jinyintan Hospital in the first week of February 2020, in Wuhan [26]. Its validation has been validated using a double-blind test, a clinically proven method. Recently, in vitro studies on chloroquine and radcivir and in combination have shown good inhibitory potential against the SARS-CoV-2 viruses [26].

As per the news reports, India-based enterprise Glenmark has secured government approval for the large-scale manufacturing and antiviral Favipiravir drug to treat SARS-CoV-2 infected patients [27]. Furthermore, molnupiravir parent drug (NHC) combination of drugs against SARS-CoV-2 viruses are recommended in a trial version of the treatment plan [28], including ritonavir/ralproveravir or ribavirin by intravenous injection in addition to the inhalation of alpha interferon [26]. Another example is the cheap and widely-available steroid drug called Dexamethasone, pushed by UK experts as a potential treatment of SARS-CoV-2 infection, with suggestions signifying great success in reducing deaths up to a third in patients showing severe symptoms, thus proving it to be a life-saving drug [29].

In May 2020, the Food and Drug Administration (FDA) of the United States was issued emergency use authorization, allowing distribution and use of remdesivir in the U.S [30], and suggested administering intravenously to treat the SARS-CoV-2 infected patients. Some reports have indicated that hydroxychloroquine and chloroquine help treat SARS-CoV-2 infected patients; however, the FDA is cautious about using both drugs [31,32]. Different patients need to be treated differently based on the symptom differentiation: the early symptom stage with stagnant lung, the middle symptom stage with pestilence, the severe symptom stage, and the final recovery stage [33]. At present, scientists from various interdisciplinary fields are working tirelessly to investigate a plethora of antiviral drugs to fight against the existing pandemic crisis posed by the COVID-19 disease (Table 1). To date, several preventive and treatment drug studies have been observed in the different clinical trial registration centers involving a variety of medicines. These include Darunavir, Corbistar, Ritonavir, Lopinavir, Camostat, Nafamostat, Umifenovir, Favipiravir, Famotidine, Ivermectin, Nitazoxanide, Tocilizumab, Corticosteroids, Sarilumab, Fluvoxamine, Bevacizumab, etc.

### 2.2. Therapeutic Models for Pulmonary Phase

An intense understanding of COVID-19 disease pathogenesis is necessary to provide a scientific foundation in developing antiviral drugs and designing vaccines. As per the recent literature developments, the progression of pathogenesis is believed to manifest in three chronological phases, including pulmonary, pro-inflammatory, and prothrombic phases.

Here, we present SARS-CoV-2-host cell interactions, key pathogenic mechanisms, and potential clinical development in the pulmonary phase and key highlights on antithrombotic therapies. In pulmonary pathogenesis, excessive cytokine production in individuals infected with SARS-CoV-2 viruses causes enhanced membrane permeability and results in pulmonary dyspnea, edema, and hypoxemia [54]. Potential therapeutics in the treatment of SARS-CoV-2 infected patients with the pulmonary phase include viral entry inhibitors, protease inhibitors, RAS inhibitors, and replication inhibitors. In particular, RAS inhibitors can be classified into angiotensin-II receptor blockers (ARBs) and inhibitors of ACE, which are involved in alleviating the RAS over-activation exerted by ACE2 deficiency in SARS-CoV-2 infected patients. However, inhibitors that target viral entry, protease, and replication are also known for direct antiviral activities via targeting specific stages of the SARS-CoV-2 life cycles.

First, RAS inhibitors such as ARBs and ACE inhibitors are generally prescribed as hypertensive drugs. Therefore, its use as a SARS-CoV-2 therapeutic raises safety concerns owing to its ability to elevate ACE2 expression, particularly in hypertensive individuals [55]. Principally, ACE2 upregulation triggered by RAS inhibition leads to the more effective entry of SARS-CoV-2 viruses; thus, there is a severe threat for the users of RAS inhibitors of becoming more vulnerable to SARS-CoV-2 infection [56]. Regardless of such health concerns, most clinicians are against the quick rejection of RAS inhibitors, particularly in high-risk individuals, including those on the verge of myocardial infarction or who have a history of heart failure. Therefore, there is a possibility of developing clinical encounters and hostile health consequences [57]. Until further development in clinical trials, clinicians are advised to continue using RAS inhibitors to achieve stable conditions in high-risk SARS-CoV-2 infected patients [58].

**Figure 1:**
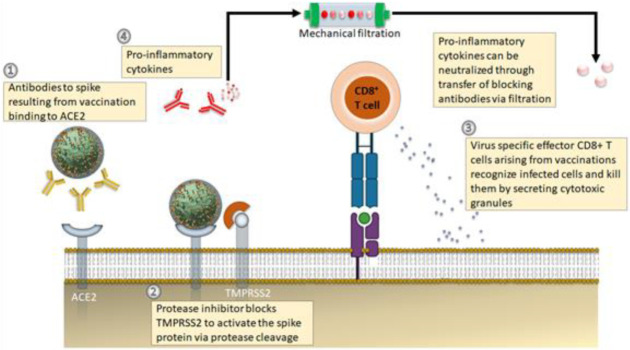
Emerging therapeutic approaches against COVID-19 disease. (**1**) Antibody-based therapeutics against the S protein (either via adoptive transfer of vaccination) to avoid progression of severe infections. (**2**) Application of protease inhibitors against serine protease (TMPRSS2) prevents cleavage of S protein. (**3**) SRS-CoV-2 virus-specific memory CD8^+^ T cells from vaccination or earlier infection. (**4**) A new treatment approach targets the symptoms of cytokine storm, wherein the blood of infected patients is passed through customized filtration columns to capture pro-inflammatory cytokines before the pureblood returns to patients. Adapted and modified from [63].

At large, RAS inhibitors seem to be advantageous to SARS-CoV-2 infected patients since they can downregulate the RAS pathway when it gets overactivated after infection, as suggested described formerly [59]. This may benefit in reducing new infections of SARS-CoV-2 viruses simply via disruption or enzymatic digestion of coronavirus spike (S) glycoprotein by transmembrane serine protease-2 (TMPRSS2), wherein involves a membrane-fusion mechanism [60]. G-protein-coupled mas receptor (MasR) agonists and ACE2-mediated stimulation of signaling through ACE2 gene delivery together with the use of angiotensin 1 to 7 have been proposed as potential immunotherapeutic approaches to encounter RAS signaling pathways, enhanced ACE2 deficiency in SARS-CoV-2 infected patients [61]. It was also shown that the ACE inhibitors suppress the expression of TMPRSS2, a key co-receptor for the entry of SARS-CoV-2 viruses [62]. Thus, it can also be a promising pathway in developing therapeutics approaches for treating SARS-CoV-2 infected patients after blocking the target ACE2 host receptors or TMPRSS2 (Figure 1).

Presently, some compounds identified that could target these molecules have been under clinical approval. For instance, applications of machine learning algorithms used to predict that JAK inhibitor called baricitinib, useful in the treatment of rheumatoid arthritis, could also be applied to inhibit endocytosis mediated by ACE2 [63]. That report also reveals clinical trial status for another potential JAK inhibitor called ruxolitinib, and it is expected to add a new treatment option. The delivery of high concentrations of ACE2 in soluble form is also considered a promising strategy to reduce the virus’s entry into host cells possibly. APEIRON is now under clinical trials with a recombinant form of ACE2 called APN01. In the case entry of SARS-CoV-2 viruses, ACE2 plays a key role, considering that neutralizing SARS-CoV-2 viruses via the administration of recombinant ACE2 protein is now being proposed as a potential therapeutic model [64]. Remdesivir and chloroquine functions at a phase post virus entry [65]. However, camostat mesylate [66], and Nafamostat mesylate [67] is an excellent inhibitor of TMPRSS2 and is under approval in several regions and countries. It is important to note that camostat mesylate is now being tested against isolated viruses from SARS-CoV-2 infected patients. It successfully prevents the SARS-CoV-2 viruses from entering into lung cells [68]. If this approach gets validation and completes clinical trials, promising repurposing of these antiviral drugs will possibly treat the SARS-CoV-2 infected patients.

At large, CD147 plays a crucial role in controlling the SARS-CoV-2 infection, including its variants and modulating their pathogenesis. The CD147 is now revealed as a universal entry receptor for the SARS-CoV-2 viruses, including its predominant variants and signaling pathway initiator, particularly cytokine storms. CD147 antibody effectively and specifically inhibit infection and block cellular entry of SARS-CoV-2 viruses and also helps in alleviating cytokine storm for almost all variants, including alpha, delta, beta, and gamma [69]. CD147 antibody named meplazumab effectively inhibits the SARS-CoV-2 virus and reduces cytokine storm by blocking the direct interactions between S glycoprotein and CD147 [70]. That report revealed that the meplazumab is appropriate in terms of tolerance and safety also recommended accelerating the recovery of severe SARS-CoV-2 infected patients. According to recent developments, the efficiency of humanized antibodies (e.g., meplazumab) have been examined against surface molecule CD147, clinical trials confirm its promising potential to treat SARS-CoV-2 infected patients [70]. Preliminary clinical trials advocate that surface molecule CD147 can also be a promising pathway to block the virus from entering the host cells, as S protein of the SARS-CoV-2 virus is also known to bind small surface molecules such as CD147 [71].

Antiviral effects of azithromycin, a macrolide antibiotic, is being presented since it reduces the viral load in hospitalized severe patients due to its ability to create interference in the course of ligand interactions with CD147 receptor [72]. Besides this, azithromycin also influences key immune parameters, including enhancing the expression of antiviral interferon and with antiviral and anti-inflammatory functions. Thus, it can be a promising therapeutic agent, but it needs validation [73].

Replication inhibitors are mainly applicable to suppressing the replication of the viral RNA genome via antagonizing the enzymatic activity such as RNA-dependent RNA polymerase (RDRP) of the SARS-CoV-2 virus. Therefore, they are mostly considered RDRP inhibitors, including favipiravir, remdesivir, and ribavirin. Favipiravir and remdesivir were specifically developed to treat influenza [74] and the Ebola virus [75], respectively. They are the nucleotide analogs helpful in hindering the functions of endogenous nucleotides in the course of viral RNA synthesis. Targets for remdesivir also include membrane protein (M protein) and RDRP inhibitors. It is the first drug approved clinically and repurposed as a potent antiviral agent for treating SARS-CoV-2 infected patients [76,77]. A well-recognized host protease inhibitor, including camostat mesylate and nafamostat, was useful in inhibiting the host TMPRSS-2 protease that restricts the entry of SARS-CoV-2 viruses into target host cells [78]. Consequently, host protease inhibitors can effectively block the virus entry; however, adverse events involve nafamostat mesylate and favipiravir in treating SARS-CoV-2 infected patients [79]. Lopinavir/ritonavir was first developed as an anti-HIV agent, now repurposed due to its inhibitory actions against protease of the SARS-CoV-2 virus [80,81].

Antithrombotic therapies are also identified as a significant approach since coagulation cascade dysregulates in SARS-CoV-2, anticoagulation can be explored in the treatment, essentially for patients with the event of venous thromboembolism (VTE) [82]. Indeed, a recent report stated approximately about 40% of SARS-CoV-2 patients hospitalized were found at high risk of VTE [83], and research data reported from extensive retrospective cohort studies for SARS-CoV-2 patients suggest that anticoagulation therapy can reduce mortality rate, especially among those patients with high severity [84]. Recent studies on the heparin-based treatment of SARS-CoV-2 have shown promising results [82,85]. This is reflected as one of the key agents to treat SARS-CoV-2 either as a therapeutic or prophylactic routine [86]. The applications of a selective antithrombin-dependent factor Xa inhibitor called fondaparinux in SARS-CoV-2 patients were reported [87]. Further studies on the application of direct oral anticoagulants in SARS-CoV-2 patients have to be studied [88,89].

Another approach to interrupt coagulation cascade via pharmacological approaches includes blockade of factor XII (FXII). It has been revealed to safeguard SARS-CoV-2 infected patients from occlusive thrombosis without any impairment in hemostasis [90,91]. Particularly, increased FXII activity was noticed in platelets isolated from the SARS-CoV-2 infected patients, which was also accounted for by the shortening of the activated thromboplastin time [92]. Likewise, serine protease inhibitors of plasmin, trypsin, and thrombin are also recognized as nafamostat mesylate, being used to treat pancreatitis and during dialysis [93], and its clinical trials are under investigation in the combination of heparin and nafamostat for SARS-CoV-2 (NCT04418128, NCT04352400).

The hypofibrinolytic state was detected in the acute respiratory distress syndrome (ARDS) and mainly being directed by the application of specific tissue-type plasminogen activators, which seems typically accountable in the transformation of plasminogen to plasmin, leads to the collapse of the cross-linked fibrin structures [94]. Indeed, infusion of tissue-type plasminogen activators has been revealed useful in severe SARS-CoV-2 infected patients [95], and current clinical trials on ARDS-related conditions are in progress with SARS-CoV-2 infected patients (NCT04357730).

Dipyridamole, an antiplatelet agent, is reported to have great therapeutic potential [96]. In addition to its antiplatelet use has also been shown multiple functions, including antiviral activity, mainly suppressing excessive inflammation and supporting mucosal healing, and preventing acute fibrosis in the lungs and other organs [97,98,99]. Treatment of SARS-CoV-2 infected patients using dipyridamole was beneficial due to its ability to prevent NETosis and promotes 3′,5′-cyclic adenosine monophosphate (cAMP) formation in neutrophils [100]. Liu et al. revealed that dipyridamole is also helpful in suppressing in vitro replication of the SARS-CoV-2 viruses and improving lung pathology in an animal model via type-I INF response [101]. Clinical trials for dipyridamole are currently under progression (NCT04391179). Alternative agent ticagrelor and antiplatelet agents also showed the ability to attenuate the formation of Neutrophil extracellular traps (NETs) [102]. Consequently, clinical trial-II on ticagrelor has been accomplished (NCT02735707, NCT04518735).

### 2.3. Anti-Inflammatory Therapeutics

The WHO advised against the clinical use of corticosteroids in treating SARS-CoV-2 infected patients, but some hospitals are still using them to treat the hyper-inflammatory symptoms [103]. However, many researchers are hopeful that corticosteroids could precisely inhibit specific pro-inflammatory pathways. One of the initial clinical reports showed elevated levels of interleukin (IL-6), which is associated mainly with the low oxygen saturation reported for SARS-CoV-2 infected patients [104], tocilizumab treatment in patients show symptoms of severe SARS-CoV-2 cases caused in better conditions, oxygen saturation, and lymphopenia conditions within a few days after treatment [105]. Similarly, in the German case study reported for 40 patients, higher levels of IL-6 are predictable for SARS-CoV-2 infected patients who will be at risk of respiratory failure. The detrimental effects of excessive levels of IL-6 are possible to be raised from both its potential to direct adverse effects on organs and arouse the immune system [106]. Thus, IL-6 receptor blocking antibodies may provide hope after challenging the hypothesis that directs anti-inflammatories pathways, thus providing great therapeutic relief [107]. According to the preliminary results of clinical trials performed for antibody therapies, it was stated to target IL-6 or IL-6R and manage appropriately. Clinical trials on mononuclear macrophages tocilizumab in fewer patients from China and France suggested that the treatment reduces the mortality and need for intensive care unit admission. Helpfully, a trial in the U.S. in a 154-patient, Tocilizumab ably reduced mortality rate in severely symptomatic patients was on ventilation [108]. The molecular mechanism of tocilizumab, binds to both the membrane and soluble forms of IL-6 receptors, thus ably suppresses the Janus kinase-signal transducer and activating transcription factors in signaling pathways [109], thus finally, the formation of inflammatory molecules [110,111]. However, IL-6-blockade may be challenging since it partly suppresses T cells’ activation. This therapy should consider disease pathophysiology without detrimental effects on organs.

## 3. Inflammatory Immunopathogenesis

Damage of lung cells after SARS-CoV-2 infections elicits local systemic and mucosal immune responses, recruits macrophages and monocytes to respond against disease progression, and also triggers cytokine storm and adaptive immune system (B and T cells). In most cases, such a response resolves SARS-CoV-2 infection. However, a dysfunction of the immune system sometimes follows, resulting in a severe condition that can distort the linings and walls of the lung’s air sacs and even cause systemic disease [112].

SARS-CoV-2 is also a cytopathic virus; it induces destruction and injury to virus-affected tissues and death of infected cells in the course of the replicative virus cycle [113]. As part of viral infection and its replication cycles in epithelial cells, it could cause severe pyroptosis and vascular leakage similar to patients with SARS-CoV [114]. This is a kind of programmed cell death manifest in highly inflammatory cells, and it can be seen in most cytopathic viruses. Pyroptosis is expected to trigger subsequent inflammatory responses. A key cytokine (IL-1β) is released during pyroptosis and elevated levels during the SARS-CoV-2 infection [2].

**Figure 2:**
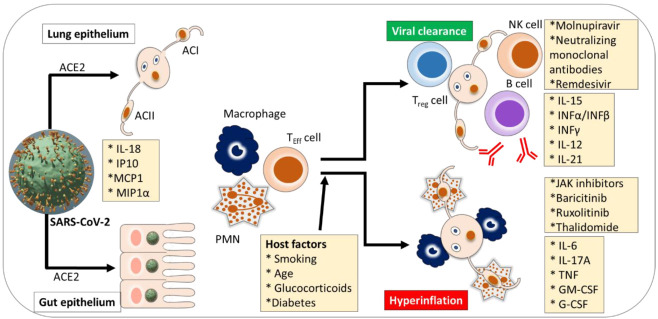
Cytokine pathogenesis of SARS-CoV-2 infection. Intensive care or appropriate treatment is recommended for hospitalized severe SARS-CoV-2 patients who exhibit elevated levels of biomarkers in blood plasma. Alveolar cell (AC), angiotensin-converting enzyme 2 (ACE2), atopic dermatitis (AD), ancestry clusters (AC1-4) granulocyte-macrophage colony-stimulating factor (GM-CSF), granulocyte colony-stimulating factor (G-CSF or GCSF), interleukin (IL), interferon (INF), monocyte chemoattractant protein 1 (MCP1), macrophage inflammatory proteins (MIP1α), natural killer (NK), polymorphonuclear granulocyte (PMN), T-effector cell (TEFF cell), tumor necrosis factor (TNF), regulatory T cell (T_reg_ cell). Adapted and modified from [116].

In most SARS-CoV-2 infected patients, recruited cells typically resolve the lung infection as immune systems respond well and infected patients recover without severe damage to lung health and other organs [63]. However, immune response dysfunction follows in several patients, which causes cytokine storm and mediates widespread inflammation in lung tissues. It can be seen in severe SARS-CoV-2 infections. Those who need intensive care or treatment in hospitals exhibit elevated levels of biomarkers in blood plasma, including interleukins (IL-2, -7, -10), interferon (IP-10), monocyte chemoattractant protein-1 (MCP1), granulocyte colony-stimulating factor (G-CSF), macrophage inflammatory protein 1α (MIP1α), and tumor necrosis factor-alpha (TNFα) [2] (see Figure 2). Also, infected individuals with severe pathology show a considerably higher count of inflammatory monocytes in peripheral blood samples than those with mild pathology [115]. These cells trigger the secretion of inflammatory cytokines that also intensify the risk of cytokine storms, including IP-10, MCP1, and MIP1α. The mechanisms associated with SARS-CoV-2 infection subvert the innate antiviral cytokine response, which is yet to be well understood. Recent research efforts show that numerous non-structural and structural proteins of viruses antagonize interferon response. Furthermore, as observed in transcriptomic analysis, dampening immune function and hyper-inflammation. Consequently, the mechanisms involved in reduced the cytokines are the most damaging. Thus, it could be assumed that TNF, IL-6, and IL-8 could be potential agents in boosting B- and T-cell response [116].

Recent reports suggest that the SARS-CoV-2 infections drive a diverse range of immune cascades, thus raising the risk of immunosuppressant agents tested in clinical trials that might vary from patient to patient and sometimes can be detrimental to some patients [117]. A diminished immune system when SARS-CoV-2 infected patients struggle off viruses can be an adverse pathophysiological state. Those infected patients had acute respiratory distress syndrome in previous times, apparently instigated by fugitive immune responses, thus resulting in cytokine storms [118,119]; clinicians are confused about the overuse of anti-inflammatory agents that helps to keep immune response in progress and avoid any collateral damages [120]. Furthermore, the upsurge in cytokines released by immune systems causes hyperinflammation in SARS-CoV-2 infection, may result in sepsis and cytokine storm, and lead to morbidity. In such cases, controlled inflammation can help avoid multi-organ failure, particularly the respiratory, hepatic, cardiac, and urinary systems. Some of the SARS-CoV-2 infected patients develop severe symptoms or organ failure eventually encounter mortality [121,122].

Both B and T cell responses were observed in blood samples for about one week from the onset of severe symptoms of the SARS-CoV-2 infection [63]. B cells response in individuals with SARS-CoV-2 disease concurrently with helper cells (T follicular) response, about one week of severe symptoms onset. Furthermore, two types of T cells include CD8^+^ T and CD4^+^ T cells play an essential role in death virus-infected cells, wherein CD4^+^ T cells are primarily accountable for controlling the production of cytokines and driving the recruitment of immune cells. If the patients with low-dose showed indication of early inflammation, the SARS-CoV-2 virus could survive even after the initial boost of immune activity. T cells are identified to battle against the SARS-CoV-2 infections; there is a chance of causing dysfunctional and metabolically exhaustion; thus, the exhausted T cells need to be substituted with fresh ones [123]. Severity progresses in SARS-CoV-2 infected patients with the simultaneous increase in the level of inflammatory cytokine may determine the exhaustion and depletion of T cell abundance.

## 4. Immune-Boosting Strategies

There is ever-growing worldwide interest in immune-boosting agents, nutrients, and yoga, which is on the rise. The changes in the immune response vary with infectious diseases, and such changes also rely on the duration of infection. Other viruses, for example, human immunodeficiency virus (HIV), hepatitis C virus (HCV), and hepatitis B virus (HBV), take several months to produce symptoms. After that, infections turn into chronic [124]. However, infections such as the influenza virus show apparent symptoms within two days of the infection, and most people recover from such virus within one week of the period [125]. However, SARS-CoV-2 infections either show no symptoms or some symptoms within four to six days of infection and, for some patients, the disease can continue for about fifteen days [126]. Subsequently, SARS-CoV-2 infection is not a chronic disease such as HIV or HBV, but also not a truly acute infection such as the H1N1 virus [127]. Various strategies could be applied to treat SARS-CoV-2 infected patients, in particular IL-7, which could promote T-cell proliferation, prevent T-cell death, and help reverse lymphopenia in severe patients [128,129].

The chronicity of the SARS-CoV-2 infection needs to be explained, especially both immune-boosting and -suppressing approaches could be potential approaches to address at different time courses [130]. Until then, the ambiguity over whether to boost or suppress the immune response led to clinical trial results contrasting to choosing appropriate treatment strategies.

After decades of R&D efforts, most of the clinical trials with anti-inflammatories resulted in failure to avoid organ damage and overcome cytokine storms. The sepsis field is now turned to address immune-activating strategies [131]. These R&D efforts are yet to deliver convincing evidence of their effectiveness. However, anti-PD1/PDL1 and IL-7 therapeutics have shown signs of usefulness and efficacy during clinical trials [132,133]. Thus, similar strategies would be effective in treating SARS-CoV-2 infected patients. Bekele et al. further revealed that IL-7 could aid to protect T cells from death, promote the proliferation of T cells, and support reversing lymphopenia in SARS-CoV-2 infected patients suffering from sepsis [133].

### 4.1. Interferon Mediated Interventions

IFN, a critical inflammatory cytokine agent that can be detected in SARS-CoV-2 infected patients, is controlled mainly by histone markers. It aids in preventing viral infections [134]. In recent studies, transcriptomic results suggested that its application may be inappropriate to control interferon response in SARS-CoV-2 infected patients [135]. That report suggests both in-vitro and in-vivo studies further reveal it to aggravate low transcripts downstream levels of both type-I and type-III interferons compared with the high level of cytokines (IL-6). Such monitoring studies proved that interferons could be considered a first-line defense against the SARS-CoV-2 infection. Thus, early improvement of interferon responses, both type-I and type-III, could be potentially adjunct patients while fighting against SARS-CoV-2 infections, allowing clearance of the virus particles before hyper-inflammation and intervention turn out to be significant challenge [63].

Furthermore, activation of IFN can be precisely modulated using epigenetic regulators, including H3K27me3, H3K4me3, and H3K9me2 [136,137]. Besides this, SARS-CoV viruses have an IFN-stimulated gene 15 (ISG-15) effector role, are essentially linked with the histone marker of ISG-15 genes on the promoters, and deviate among a range of viruses [138,139]. IFNs are also useful in controlling certain cancers (Borden 2019) and hepatitis C [140]. IFN-α combined with anti-viral drug ribavirin was identified as the backbone for HCV treatment up to 2014. SARS-CoV-2 viruses were identified to interact characteristically with the ubiquitin-like ISG-15, a critical innate-immune controller of host cells. Additionally, preferential cleavage of ISG-15 via protease of the virus (PLpro) may weaken the signaling pathways of type-I IFN, which is a vital component in most of the antiviral responses, and IFN responsive factor-3 (IRF3) [141]. The PLpro is essential for the formation of appropriate function replicase complex and promoting viral spread. Several key proteins of the SARS-CoV-2 virus, including N protein, structural proteins, and some other accessory proteins such as ORF8 and ORF6, were recently established as potential inhibitors of the type-I IFN pathway [142]. However, recent clinical studies have demonstrated an absence of noticeable type-I IFN among most of SARS-CoV-2 infected patients [143].

CD4^+^ T cells that are reactive to M protein are identified as multifunctional accompanied by increased levels of IL-2, TNF-α, and IFN-γ, consequently S protein and ultimately CD4^+^ T cells reactive to N proteins [144]. Furthermore, CD8^+^ T cells were recently studied in SARS-CoV-2 for IFN-γ production. However, levels of CD8^+^ T cells remain lower than that of CD4^+^ T cells [145]. Another important clinical study suggested that in response to S or N proteins, the level of IFN-γ remains higher in SARS-CoV-2 infected patients with mild infections than severe ones [146].

A recently published report showed that triple combination of ribavirin, ritonavir/lopinavir, and IFNβ-1b was found to be effective and safe in patients with mild symptoms compared to ritonavir/lopinavir in concern to control symptoms, promote viral shedding, and shorten the hospital stay [147]. However, a clinical trial performed with 127-person showed evidence supporting the importance of interferons in decreasing the course of SARS-CoV-2 infection by simply using repurposed drugs ritonavir, ribavirin, and lopinavir in combination with IFNβ [147]. Therefore, a type I interferon (IFNβ) is a solitary immune-boosting approach that has to be further investigated in the large-scale trial in combination with nominated anti-viral drugs [148]. In addition, a type III interferon (IFNλ) in patients has to be tested with those mild or moderate symptoms [149]. Since IFNλ receptors are expressed on epithelial cells, some normal cells thus need to be tested for any complications related to cytokine production and organ-damaging, as reported previously [150]. Furthermore, previous studies showed no advantage from IFN-α/β in severe patients with SARS and MERS viruses [151,152,153]. In contrast, IFNs show noticeable adverse symptoms, including flu-like, headaches, gastrointestinal issues, and allergic reactions. Further studies are required to validate the data and evaluate the clinical uses and potential toxicity concerns of IFNs.

### 4.2. Restoring Distressed T-Cells

Other strategies have reflected advantages to achieve immune-boosting and restoring exhausted T-cells. Often T cells cannot keep up with the vitality demanded during prolonged battles against infection since, by time course, they get a decline in their function through a series of processes as reported for lymphocytic choriomeningitis virus [154]. The report has shown that patients with chronic HIV, HBV, or HCV infections can cause exhaustion of T-cells, though T-cells could keep the infection on a smoldering level. However, they seem powerless to eradicate infection [155]. An important marker for T cell fatigue receptor is that the programmed death (PD1) gets upregulated in infected cells and dampens the normal functioning of T cells. However, antibodies targeting PD1 and its ligands programmed death-ligand (PDL1), cytotoxic T-lymphocyte-associated protein-4, and some other inhibitory surface receptors can transform SARS-CoV-2 treatment performed simply by reenergizing the immune response [156]. Recently it was evaluated whether activation of human macrophage, astrocytes, microglia, brain endothelial cells, and neurons using safe level concentrations of ethanol exposure ably alters expression of PD-1/PD-L1 [157]. However, PD1-targeting antibodies showed good efficacy, demonstrating that the PD1 inhibition is applicable in human diseases, wherein PD1-blocking antibodies assist to increases the active T-cell population. Thus, it helps in chronic infection such as HIV [158]. Trials examining applications of anti-PD1 treatments in SARS-CoV-2 are yet to be completed [159]. Another approach emphasizes the significance of providing an energy boost to T-cells using antioxidants and restoring immune balance in SARS-CoV-2 infected patients, hoping that antioxidant compounds such as N-acetylcysteine could restore T-cell function [160]. Those clinical trials have to examine whether repurposed strategies for immune-boosting are effective, safe, or address hyper-inflamed states in patients.

## 5. Tracking Immune Status

Severe SARS-CoV-2 infected patients have to be timely treated on the basis of their immune status. However, there is a need to define how this could be achieved [161]. According to the current WHO guidelines, which are often recommended to arrange trials based on exclusion and inclusion criteria, infected individuals transition from mild to severe or need ventilator support [162]. However, the immune status reflects the level of healthcare needs for the particular patient instead of the underlying immune status or biological processes. Therefore, considering several biomarkers could recognize the patients who are mostly expected to respond positively to a specified immunomodulating agent [163]. Most biomarkers are based on a single protein being developed to stratify sepsis individuals [164]. However, it is challenging to discern or validate as SARS-CoV-2 virus biomarkers. There is a need for specific functional assays to identify or count the active T cells in the blood samples [165]. Integrating several key biomarkers into a single signature can be a promising approach. Metabolomic and proteomic characterization of serum samples was reported by a research group led by Tiannan Guo [166], wherein blood samples from 46 SARS-CoV-2 infected patients and 53 uninfected individuals were analyzed using mass spectrometry. That report suggests that proteomic and metabolomic fingerprints can be prepared and validated in other cohort studies, including main 22 proteins and seven other metabolites are proposed to evaluate the severity of the disease. Another report similarly suggested a broad approach-based signature, wherein samples were collected from early hospitalized cases and used to identify potential proteins about 27 potential biomarkers for their increasing or decreasing levels. In Table 2, an important pro-inflammatory signaling pathway is also discussed, which expresses differentially both downstream and upstream of IL-6, is in agreement with WHO severity grade provided for the SARS-CoV-2 infection [167]. Understanding the immune response at a molecular and functional level for SARS-CoV-2 infections is vital, which is not either acute or chronic infection. Such studies could also be indispensable to be prepared against future pandemic viruses [168] and also help in developing promising vaccines against SARS-CoV-2 viruses [169].

Confronting the challenge brought by the COVID-19 pandemic crisis to public health, we still lack the ways to explain the differences among the immune response and symptoms of infected patients. The fundamental key is a comprehensive understanding and significance of SARS-CoV-2 infected patients for their diverse immune response or immune indicators to inflammatory changes from initial onset to the end. There is scope to provide new approaches to overcome the existing pandemic by performing dynamic analysis of lymphocyte subsets, leukocyte classification, and cytokines. It was suggested that the total counts of lymphocytes, leucocytes, and eosinophils in SARS-CoV-2 infected patients decline. However, there was a significant difference for monocytes and neutrophils compared to non-infected individuals [203]. That report further reveals that the counts of leukocytes and neutrophils increase, eosinophils and lymphocytes continue to decline, and monocytes count remains stable (particularly in severe SARS-CoV-2 infected patients, which is partly consistent with another report [1]. The increase of monocytes in SARS-CoV-2 infected patients compared to healthy individuals is caused by inflammation-triggered monocyte infiltration and activation [204]. There is an observation on the increase in levels of leukocytes and neutrophils, mainly in severe patients and those with secondary infections. It can be explained reasonably as downregulation of ACE2 occurs on the onset of the COVID-19 disease and causes infiltration of neutrophils, which further cause tissue damage and then manifests into venous thrombosis [205]. Furthermore, a recent report showed a significant decrease in eosinophil count compared to the healthy control. Therefore, involvement of eosinophils is expected in immune response and viral clearance [206].

In the recent literature, we noted an interesting and perhaps controversial concern about the existing pandemic and still considerable uncertainty about the success of the current therapeutic interventions on the immune response [100]. This would interfere with the longitudinal detection approaches available so far to examine the immune status of SARS-CoV-2 infected patients. Firstly, virus-targeting anti-viral drugs were commonly applied for SARS-CoV-2 infected patients, including lopinavir or ritonavir and ribavirin and α-IFN and chloroquine as host-targeting drugs, were reported in a previous report [1]. These drugs were initially used as protease inhibitors for several other viruses and also investigated for SARS and MERS infections. However, chloroquine was found to be an outstanding candidate since it acts as an immune modulator and suppresses the SARS-CoV-2 infection [207]. Several anti-viral agents are underpinning clinical trials and are yet to be confirmed for their efficiency against SARS-CoV-2 viruses and their effect on the immune response [208]. Recently it was proposed that α-IFN regulates monocyte-derived macrophages, NK cells, and T cells in SARS-CoV-2 infected patients. However, the immune response triggered by α-IFN is still unclear [209]. The recent report on the effects of chloroquine suggests promising results in treating SARS-CoV-2 infected patients; efficacy and impact on immunity must be in the final stage of clinical trials [210,211,212]. So far, we are on the verge of rapid progress in evaluating the dynamic changes between the human host and SARS-CoV-2 viruses. It is under current treatment through monitoring immune response and a well-thought-out guide to choosing the right drugs optimal medication time. Second, an appropriate selection of therapeutic interventions is quite challenging to apply firmly, followed by a new variant of SARS-CoV-2 virus for both mild and severe patients. However, it would be reasonable to successfully treat SARS-CoV-2 infected patients based on the approved evaluation criteria and treatment principles. Therefore, this report suggests validating the effects of different treatment options, preparing the database for several severity groups among SARS-CoV-2 infected patients, and matching the results using a continuous monitoring approach for immune indexes. Furthermore, the biomarkers monitored by clinical teams could more accurately reflect the clinical significance and immune status of SARS-CoV-2 infected individuals.

## 6. Future Perspectives

Researchers have made enormous efforts to improve our understanding of the pathogenesis of the SARS-CoV-2 infections and find therapeutics to relieve the existing pandemic crisis. After the beginning pandemic, most research was focused on viral proteins. The basis for this was previous developments on SARS and MERS, since they share identical genomic features and some of the same viral proteins. Therefore, repurposing previous drugs was the ideal choice, as it can save the time required for drug discovery, and its safety and efficacy needed to be clinically validated. However, some satisfactory results from the clinical trials for repurposing drugs were reported. This review highlights recent advancements in emergent interventional potential therapeutic targets and clinical strategies based on the target host and virus.

Due to structural proteins of the SARS-CoV-2 virus, S protein seems to be the most promising target to develop direct antiviral agents, and current developments on crystal structure play a significant role in blocking the replication cycle of the SARS-CoV-2 virus. The common structural proteins among coronaviruses may assist in determining the results of the antiviral strategies. Some of the nonstructural proteins of the target virus are vital in both the replication cycle of the target virus and understanding virus-host interactions, which can be promising indirect targets for developing antiviral therapeutics. Therefore, precise knowledge about structures of target proteins and the clinical pathogenesis of this infection progression may benefit from revealing potential therapeutic agents needed to encounter SARS-CoV-2 infections. However, with an insignificant accomplishment for potential drugs targets/emerging therapeutics in battling SARS-CoV-2 infections, we suggest that most repurposed or newly developed drugs had only minor success in their preclinical trials. Although few targeted antiviral drugs were fortunately entered in the clinical trials, some of them also failed in the final phase of the clinical trials (including some of the vaccines), which may be reconsidered for further investigation by researchers. There is still a huge information gap among new targets, potential drug discovery, and clinical trials. Several new strategies should be deliberated to address the existing gap in the near future and to anticipate breakthrough research achievements combating the COVID-19 pandemic by focusing on R&D efforts for most of the potential drug targets in the current drug development programs, strengthening collaboration among various disciplines, and monitoring mutation event in long-term is a must.

In consideration of host immune response, both B and T cell immune responses to SARS-CoV-2 infections is remained poorly understood. Some of the recent studies suggest that immune response, particularly an aggressive one, leads to immunopathology, whereas other reports indicated that the mechanism of T cell exhaustion or dysfunction is also marked [213,214,215]. Examinations showed high levels of viral particles in the respiratory tract and some other tissues [119], representing an incompetent immune response. Nevertheless, asymptomatic or mild patients who do recover from infections had evidence of SARS-CoV-2-specific T cell memory [216]. Antibodies specific to SARS-CoV-2 are found in recovering patients, and individuals are under treatment with plasma therapy [217,218].

Conversely, SARS-CoV-2 infected patients under intensive care units have virus-specific antibodies, raising questions concerning why those patients cannot manage the disease. At large, some of the previous studies have stated observations on few patients or small cohorts’ studies, and thus further comprehensive studies with deep immune profiling of hospitalized SARS-CoV-2 infected patients are needed. Such revelations would address the critical questions, including common immune dysfunction profiles in severely ill patients. Such developments will also aid in preparing a framework of testing key therapeutic agents to inhibit, enhance, or otherwise engineer the immune response in SARS-CoV-2 infected patients.

A significant implication is that the potential to combine immune features with the severity of disease at the sampling time along in the course of disease severity changes over a progression. Application of correlative examination allows one to observe relationships among characteristics of the diverse immunotypes, comorbidity factors, and clinical aspects of the SARS-CoV-2 virus. After integrating several immune features using comprehensive clinical data, temporal changes, and severity scores, one can develop a computational model that integrates and connects the phenotype of patient immune response with the severity of the disease. This approach may allow us to connect dots to integrate immune signatures data to clinically measurable disease features. In the future, comprehensive data sets on circulating inflammatory mediators and immune cell types for most patients will improve understanding of immune responses. However, such findings may incite the idea of modifying clinical treatments or immune-based future clinical trials to individuals whose immunotype shows potential benefits. Respiratory viral diseases cause clinical pathology due to an immune response that is either too weak with virus-induced pathology or too strong with immunopathology [135]. Recent developments in the scientific literature suggest that the immune response of severe SARS-CoV-2 infected patients may fall across the range of immune response patterns that exist as diverse immunotypes associated with clinical features, severity, and temporal changes in immune response and pathogenesis. This account highlights the significance of immune response and developing an integrated framework to link clinical pathology with the disease severity.

## 7. Conclusions

Herein, we present the significance of understanding the severe symptoms in SARS-CoV-2 infected patients and the need for potential anti-inflammatory measures, which is of great interest since hyper inflammation enhances the risk and severity of the disease. Strategic investigations are also needed to track immune responses and explain how some infected people recover naturally and others did not. Such knowledge would also lead us to develop effective vaccines and therapeutics. Based on recent developments in the literature, we believe that deficiency of type-I IFN is a sign of severe SARS-CoV-2 infected patients. Thus, we infer that the severity in SARS-CoV-2 infected patients can be comforted after considering fulfilling IFN deficiency via administration of IFN and addressing excessive inflammation via appropriate anti-inflammatory therapies that target TNF-α or IL-6. However, this hypothesis needs further testing and elucidating regarding how SARS-CoV-2 viruses cause cascade pathways in the immune system. Interdisciplinary efforts from expertise in microbiology, medicine, public health, pharmacology, and information technology and the contribution of environmental scientists are immediately needed to battle against the prevailing crisis. Continuous monitoring of immune responses onset of SARS-CoV-2 infection is still challenging considering both diverse patterns and complexity. Thus, comprehensive revelations on an immune response would be a practical approach to challenge the current pandemic crisis, the emergence of SARS-CoV-2 variants, and future pandemic pathogens.

## Figures and Tables

**Table 1:** Mechanism of types of drugs and therapies useful in the treatment of SARS-CoV-2-infected patients [3,27,32].

Antiviral Agents	Mechanism of Action	Reference
Lopinavir–ritonavir	It is used as a protease inhibitor, inhibition of 3-chymotrypsin-like protease.	[34]
Molnupiravir	It is the first oral antiviral drug, highly effective in the treatment of SARS-CoV-2 infected patients.	[35]
Glucocorticoids	It is used for the suppression of immune and inflammatory responses.	[36]
Nafamostat	It acts as a serine protease inhibitor that helps in blocking the entry of SARS-CoV viruses.	[37]
Chloroquine or hydroxychloroquine	This drug creates interference with ACE2 and blocks virus invasion. It does not allow to increase of endosomal pH, which is required for virus fusion and helps in mild immune suppression.	[38]
Thalidomide	This agent has multiple benefits, including reducing inflammatory cell infiltration, relieving cytokine storm, reducing lung damage, and avoiding pulmonary interstitial fibrosis.	[39]
Famotidine	It acts as H_2_ receptor antagonist.	[40]
Leflunomide	It helps in the inhibition of virus replication.	[41]
Tocilizumab	It acts as a blockade for IL-1 receptors. It also involves downstream signaling pathways. It can reduce mortality and pathology.	[42]
Anakinra	It acts as a blockade for IL-1 receptors and involves downstream signaling pathways.	[43]
Bevacizumab	It helps in reduces vascular permeability and decreases the amount of fluid that enters the lung tissues.	[44]
Umifenovir	It is a small indole-derivative, which has antiviral properties. It can effectively block trimerization of S glycoprotein of SARS-CoV-2 virus.	[45]
Baricitinib	It acts as a Janus kinase (JAK) inhibitor; it can blockade viral entry by inhibiting AAK1. It also helps in immune suppression.	[46]
Arbidol (Ruxolitinib)	It acts as JAK inhibitor and helps in immune suppression.	[47]
Nitazoxanide	It involves blocking the maturation of the nucleocapsid N protein.	[48]
Stem cell therapy	It helps in suppressing inflammation and proviral silencing. Decreases inflammation indicators (lowers IL-6 and CRP levels)	[49,50]
Convalescent plasma therapy	It promotes the elimination of viruses through specific antibodies.	[51]
Neutralizing monoclonal antibodies	Pathogen-specific antibodies involve in antibody effector activity and allow the neutralization of pathogens.	[52]
Fc-engineered antibody	Application of monoclonal antibodies optimized Fc domains with the optimal Fc-effector function and better clinical effectiveness against the SARS-CoV-2 infection.	[53]

**Table 2:** List of potential biomarkers of SARS-CoV-2 infection.

Pathway	Biomarkers	Comment	Reference
Hematological	White blood cells (WBC) count	Elevated levels. WBC count is significantly correlated with severity in SARS-CoV-2 infected patients. Higher levels of WBC suggest the need for more attention during the treatment of SARS-CoV-2 infected patients.	[170]
	Lymphocyte count (LC)	Decreased levels. Recent findings show that absolute lymphocyte count remains lower in severe SARS-CoV-2 infected patients.	[171]
	Neutrophil count (NC)	Elevated levels. Increased levels of circulating neutrophils are characteristically realized in SARS-CoV-2 infected patients.	[172]
	Monocyte-to-lymphocyte ratio (MLR)	Elevated levels. Elevated MLR and higher mortality among SARS-CoV-2 infected patients.	[173]
	Neutrophils-to-lymphocyte ratio (NLR)	Elevated levels. NLR at the initial stage is lower and has a significant prognostic value in SARS-CoV-2 infected patients as compared with influenza and syncytial virus.	[174]
	Mean platelet volume (MPV)	Elevated levels. MPV found significantly higher levels in severe patients compared to mild patients.	[175]
	Red cell distribution width (RDW)	Elevated levels. RDW levels can be a potent predictor for the severity of SARS-CoV-2 infected patients and the probability of mortality.	
	Monocyte distribution width (MDW)	Elevated levels. MDW can be performed using cell count analyzers and can be reliable marker of sepsis for early-stage diagnosis.	[176]
	Lymphocyte subsets (neutrophilia, lymphopenia, T-helper (CD4^+^) and T-cytotoxic (CD8^+^))	Lymphocyte subsets have a correlation with the severity and outcome of the disease in SARS-CoV-2 infected patients.	[177]
Inflammation	Elevated IL-2, IL-6, IL-8, IL-10 levels	Elevated interleukin levels are highly associated with severe SARS-CoV-2 infected patients.	[178]
	Procalcitonin (PCT) level	Elevated levels. Serial measurement of PCT levels help to predict the prognosis of SARS-CoV-2 infected patients.	[179]
	Serum amyloid A (SAA)	Elevated levels. Significantly higher SAA levels in SARS-CoV-2 infected patients with non-survivors and severe disease.	[180]
	Erythrocyte sedimentation rate (ESR)	Elevated levels. Significantly higher ESR levels were found in severe patients than non-severe patients.	[181]
	C-reactive protein (CRP)	Elevated levels. High serum levels of CRP contribute to the SARS-CoV-2 infection progression.	[182]
	Ferritin	Elevated levels. Ferritin is iron-containing an intracellular blood protein.	[183]
	Sphingosine-1-phosphate	Decreased levels. A decrease in serum sphingosine-1-phosphate levels can be a predictor of severity in SARS-CoV-2 infected patients.	[184]
Coagulation	Elevated fibrin/fibrinogen (FIB) degradation factor	Elevated fibrinogen (FIB) can be one of the important indicator as fibrinolysis abnormalities and coagulation among SARS-CoV-2 infected patients.	[185]
	D-dimer levels	Elevated level of D-dimer is one of the important measure can be used to detect thrombosis among SARS-CoV-2 infected patients.	[186]
	Prothrombin time (PT)	The admission prothrombin time (PT) significantly higher among non-survivor SARS-CoV-2 infected patients.	[187]
	Prothrombin time activity (PT-act)	Prothrombin time activity (PT-act) higher among non-survivor SARS-CoV-2 infected patients.	[185]
	Partial thromboplastin time (aPTT)	Anticoagulants based therapy required in SARS-CoV-2 infected patients for aPTT prolongation.	[188]
	PT-international normalized ratio (INR)	INR in SARS-CoV-2 infected patients were found higher than healthy individuals and were also found higher in the patients with thrombotic disease than that of without this disease.	[189]
	Liver fibrosis index (FIB-4)	This is one of the important test widely used to detect a stage of fibrosis and to monitor chronic liver diseases, such as chronic viral hepatitis, HCV/HIV co-infection, as well as metabolic-associated fatty liver disease.	[190]
Necrosis	Lactate dehydrogenase (LDH)	Elevated levels. LDH is a marker of various inflammatory states, e.g., infections including SARS-CoV-2 infection, myocardial infractions, and malignancies.	[191]
Liver injury	Alanine transferase (ALT)	Elevated levels. Evidence of liver injury in SARS-CoV-2 infected patients with abnormal ALT levels is based on the severity of the disease.	[192]
	Aspartate transaminase (AST)	A high AST/ALT ratio can indicate a worse state and mortality among SARS-CoV-2 infected patients.	[193]
	Serum total bilirubin (STB)	Elevated levels. SARS-CoV-2 infected patients with elevated STB levels had higher mortality.	[194]
	Unconjugated bilirubin (UCB)	Elevated UCB levels SARS-CoV-2 infected patients can predict the severity of disease and probability of mortality.	[194]
	Gamma-glutamyl transferase (GGT)	Elevated GGT levels indicate the severity and are positively correlated with a prolonged hospital stay.	[195]
Cardiac injury	D-dimer	Early-stage elevated levels of D-dimer levels among SARS-CoV-2 infected patients indicate pulmonary intravascular coagulation.	[195]
	Homocysteine	Elevated homocysteine levels are a potential predictor of cardiovascular risk in SARS-CoV-2 infected patients.	[196]
	N-terminal pro-brain natriuretic peptide (NT-pro-BNP)	Elevated NT-proBNP levels is associated with high mortality risk in SARS-CoV-2 infected patients even with no history of heart failure.	[197]
	Serum cardiac troponin (cTn)	Elevated cTn is a strong indication of myocardial injury in SARS-CoV-2 infected patients.	[198]
Muscular injury	Creatine kinase	Elevated CK levels are a potential biomarker of muscle damage, possibly correlated with more severe SARS-CoV-2 infection.	[199]
	Myoglobin	Myoglobin is oxygen and iron-binding protein that enables oxygen storage in cardiac and skeletal muscles. Significantly elevated levels of myoglobin were observed in SARS-CoV-2 infection with severe and death groups.	[200]
Renal	Serum creatinine	Serum creatinine levels were significantly higher in the severe and mortality groups than recovery groups.	[201]
Organ failure	MR-pro-ADM	Elevated MR-proADM can be an emergent prognostic factor in evaluates deteriorating state SARS-CoV-2 infected patients.	[202]
