# Correction: Saratale et al. Significance of Immune Status of SARS-CoV-2 Infected Patients in Determining the Efficacy of Therapeutic Interventions. *J. Pers. Med.* 2022, *12*, 349

**DOI:** 10.3390/jpm12060967

**Published:** 2022-06-14

**Authors:** Ganesh Dattatraya Saratale, Han-Seung Shin, Surendra Krushna Shinde, Dae-Young Kim, Rijuta Ganesh Saratale, Avinash Ashok Kadam, Manu Kumar, Ali Hassan Bahkali, Asad Syed, Gajanan Sampatrao Ghodake

**Affiliations:** 1 Department of Food Science and Biotechnology, Dongguk University-Seoul, 32 Dongguk-ro, Ilsandong-gu, Goyang-si 10326, Gyeonggi-do, Korea; gdsaratale@dongguk.edu (G.D.S.); spartan@dongguk.edu (H.-S.S.); 2 Department of Biological and Environmental Science, Dongguk University-Seoul, 32 Dongguk-ro, Ilsandong-gu, Goyang-si 10326, Gyeonggi-do, Korea; shindesurendra9@gmail.com (S.K.S.); sbpkim@dongguk.edu (D.-Y.K.); 3 Research Institute of Biotechnology and Medical Converged Science, Dongguk University-Seoul, 32 Dongguk-ro, Ilsandong-gu, Goyang-si 10326, Gyeonggi-do, Korea; rijutaganesh@gmail.com (R.G.S.); avikadam2010@gmail.com (A.A.K.); 4 Department of Life Science, Dongguk University-Seoul, 32 Dongguk-ro, Ilsandong-gu, Goyang-si 10326, Gyeonggi-do, Korea; manukumar007@gmail.com; 5 Department of Botany and Microbiology, College of Science, King Saud University, P.O. Box 2455, Riyadh 11451, Saudi Arabia; abahkali@ksu.edu.sa (A.H.B.); assyed@ksu.edu.sa (A.S.)

## Errors in Figures and Table Caption

In the original publication [1], there were mistakes in the legends for Figure 1, Figure 2, and Table 1. We failed to mention the citation number in these legends. The correct legends appear below.

Figure 1. Emerging therapeutic approaches against COVID-19 disease: (1) antibody-based therapeutics against the S protein (either via adoptive transfer of vaccination) to avoid progression of severe infections; (2) application of protease inhibitors against serine protease (TMPRSS2) prevents cleavage of S protein; (3) SRS-CoV-2 virus-specific memory CD8+ T cells from vaccination or earlier infection; (4) a new treatment approach targets the symptoms of cytokine storm, wherein the blood of infected patients is passed through customized filtration columns to capture pro-inflammatory cytokines before the pureblood returns to patients. Adapted and modified from [63].

Figure 2. Cytokine pathogenesis of SARS-CoV-2 infection. Intensive care or appropriate treatment is recommended for hospitalized severe SARS-CoV-2 patients who exhibit elevated levels of biomarkers in blood plasma: alveolar cell (AC), angiotensin-converting enzyme 2 (ACE2), atopic dermatitis (AD), ancestry clusters (AC1-4) granulocyte-macrophage colony–stimulating factor (GM–CSF), granulocyte colony–stimulating factor (G–CSF or GCSF), interleukin (IL), interferon (INF), monocyte chemoattractant protein 1 (MCP1), macrophage inflammatory proteins (MIP1α), natural killer (NK), polymorphonuclear granulocyte (PMN), T-effector cell (TEFF cell), tumor necrosis factor (TNF), regulatory T cell (Treg cell). Adapted and modified from [116].

Table 1. Mechanism of types of drugs and therapies useful in the treatment of SARS-CoV-2-infected patients [3,27,32].

The authors apologize for any inconvenience caused and state that the scientific conclusions are unaffected. These corrections were approved by the Academic Editor. The original publication has also been updated.
